# Sex-Specific miRNA Differences in Liquid Biopsies from Subjects with Solid Tumors and Healthy Controls

**DOI:** 10.3390/epigenomes7010002

**Published:** 2023-01-10

**Authors:** Elena Tomeva, Ulrike D. B. Krammer, Olivier J. Switzeny, Alexander G. Haslberger, Berit Hippe

**Affiliations:** 1 HealthBioCare GmbH, A-1090 Vienna, Austria; 2 Department of Nutritional Science, University of Vienna, A-1090 Vienna, Austria

**Keywords:** miRNA, DNA methylation, cancer, solid tumor, epigenetics, sex dimorphism, liquid biopsy, biomarker, CRC

## Abstract

Dysregulation of epigenetic mechanisms has been recognized to play a crucial role in cancer development, but these mechanisms vary between sexes. Therefore, we focused on sex-specific differences in the context of cancer-based data from a recent study. A total of 12 cell-free DNA methylation targets in CpG-rich promoter regions and 48 miRNAs were analyzed by qPCR in plasma samples from 8 female and 7 male healthy controls as well as 48 female and 80 male subjects with solid tumors of the bladder, brain, colorectal region (CRC), lung, stomach, pancreas, and liver. Due to the small sample size in some groups and/or the non-balanced distribution of men and women, sex-specific differences were evaluated statistically only in healthy subjects, CRC, stomach or pancreas cancer patients, and all cancer subjects combined (*n* female/male—8/7, 14/14, 8/15, 6/6, 48/80, respectively). Several miRNAs with opposing expressions between the sexes were observed for healthy subjects (miR-17-5p, miR-26b-5p); CRC patients (miR-186-5p, miR-22-3p, miR-22-5p, miR-25-3p, miR-92a-3p, miR-16-5p); stomach cancer patients (miR-133a-3p, miR-22-5p); and all cancer patients combined (miR-126-3p, miR-21-5p, miR-92a-3p, miR-183-5p). Moreover, sex-specific correlations that were dependent on cancer stage were observed in women (miR-27a-3p) and men (miR-17-5p, miR-20a-5p). Our results indicate the complex and distinct role of epigenetic regulation, particularly miRNAs, depending not only on the health status but also on the sex of the patient. The same miRNAs could have diverse effects in different tissues and opposing effects between the biological sexes, which should be considered in biomarker research.

## 1. Introduction

The occurrence of many diseases, for instance, cancer, cardiovascular, autoimmune, and neurodegenerative diseases, has an altered sex ratio [1]. The sex-specific susceptibility to certain disorders could be partly explained by basic genetic differences (sex chromosomes) and gonadal hormones [1,2], as well as differences in risk factors or disease prevention strategies for women and men [3]. The realization that a sex bias in disease exists has led to the exploration of the possible biological processes that are involved in sex-related disease development. While the human genome between females and males varies in sequence only regarding the Y chromosome, differences between the two sexes are additionally driven by sex-specific gene expression regulators [4]. Epigenetic mechanisms for example, such as DNA methylation and microRNAs (miRNAs) play an important role in the gene regulatory machinery. Dysregulation of these epigenetic pathways has been linked to various human pathologies [5,6] and it has been especially recognized as a crucial driver of carcinogenesis [7].

DNA methylation occurs primarily in CpG dinucleotides and describes the covalent binding of a methyl group to the fifth position of the cytosine. This mechanism regulates gene expression in normal cells and is also directly involved in disease development [8]. Further important epigenetic gene regulators are a special class of small non-coding RNAs, the miRNAs. Through their dynamic interaction with messenger RNA (mRNA), they are involved in post-translational regulation and play an important role in various biological processes, such as regulating energy metabolism, cell senescence, and apoptosis [9,10,11]. For instance, miR-92a has been shown not only to induce cell proliferation in cancer cells but it is involved in apoptosis as well [12,13]. Although miR-17 belongs to the same miRNA cluster as miR-92a (miR-17-92 cluster), a functional antagonism between these two miRNAs has been suggested, whereas miR-17 acts as a suppressor of the oncogenic effects of miR-92a [14]. Furthermore, the complexity of miRNA-mediated regulation has been shown for cyclin-dependent kinase inhibitor 1A (CDKN1A), a key regulator of cell cycle progression. Several different miRNAs directly target the three prime untranslated region (3′UTR) of *CDKN1A*, leading to cell growth and progression and are thereby involved in oncogenesis [15]. In order to meet their increased energy demands during growth, the glucose metabolism of cancer cells is often accelerated through higher levels of glucose transporters (GLUT). MiR-195, miR-186, and miR-22 directly target the 3′UTRs of several *GLUT*s and are, therefore, involved in cancer cell energy metabolism [10].

Since epigenetic changes occur early in tumor development, and these alterations are reversible, recent studies have mainly focused on epigenetic biomarkers. Moreover, epigenetic alterations that are driven by carcinogenesis have been detected in so-called liquid biopsies [16,17], which, in contrast to tissue biopsies, are minimally invasive sampling methods of body fluids. Additionally, blood-based liquid biopsies could better reflect the heterogeneity of tumors and have the potential to be simple tools for disease diagnosis and real-time monitoring [18]. DNA methylation of specific regions or miRNAs could serve not only as tools for early disease diagnosis but also as therapeutic targets [19]. However, sex-related differences in DNA methylation or miRNA regulation have been reported [5,20] and could thereby be involved in sex-biased disease outcomes. Yet, this sex bias is seldom considered in epigenetic biomarkers research. Moreover, the mechanistic pathways behind sex-related cancer development are not fully understood. These epigenetic variations should be acknowledged, especially in clinical trials, in order to develop effective prevention and therapeutic strategies that are applicable to both sexes. Such sex-adjusted biomarkers could improve disease diagnosis and treatment outcomes.

To highlight the sex bias in epigenetic biomarkers, we analyzed a subset of data that were collected from a recent study [21] including liquid biopsy samples from healthy controls and patients with bladder, brain, colorectal, lung, stomach, pancreas, and liver cancer. We investigated whether sexual dimorphism is present in the collected data for miRNA expression and cell-free DNA methylation. By delving deeper into the epigenetic differences between females and males, we aim to further elucidate the sex bias in cancer diseases and underline the importance of personalized healthcare. Here, the term sex refers to the biological sex that was assigned at birth in binary form and does not consider gender identity. The study participants were classified as either female or male at the time point of sample collection. 

## 2. Results

### 2.1. Differences in miRNA Expression between Female and Male Participants

**Figure 1:**
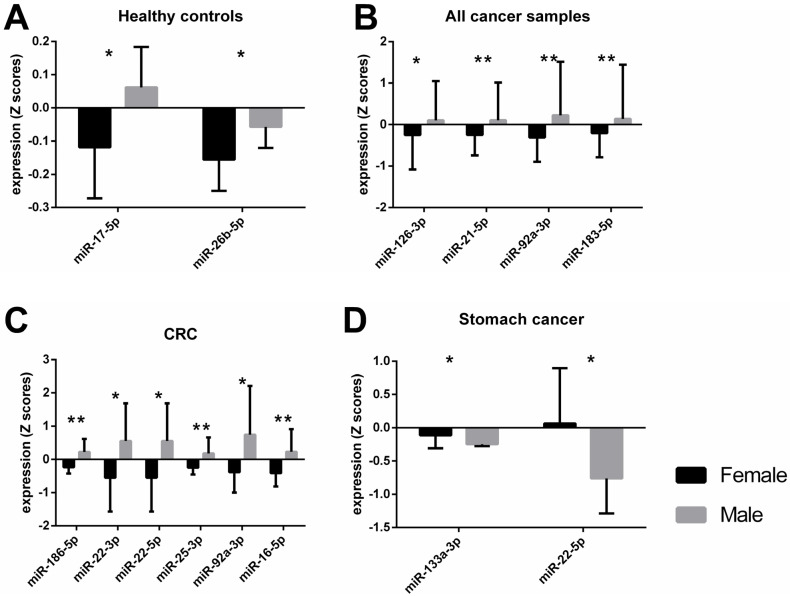
Different miRNA expression levels between female and male participants when tested across different groups after adjustments for covariates. Bars represent the mean value with standard deviation (SD). The asterisks indicate a statistically significant difference between the groups * *p* < 0.05, ** *p* < 0.01. A healthy control group, *n* female = 8, *n* male = 7 B all cancer samples, *n* female = 48, *n* male = 80 C colorectal cancer group, *n* female = 14, *n* male = 14 D stomach cancer, *n* female = 15, *n* male = 8.

An independent *t*-test showed significant differences between male and female subjects in the healthy control group for miR-17-5p and miR-26b-5p levels (Figure 1A). When miRNA expression data for male and female subjects with solid tumors, irrespective of cancer type was analyzed with a *t*-test, significant differences were observed for miR-126-3p, -21-5p, -92a-3p, -183-5p, and -16-5p (Table 1). After an adjustment for cancer type and stage, body mass index (BMI), and age, these differences were still present, with the exception of miR-16-5p. Since the distribution of female and male participants differed greatly in each cancer type group (Table 2), we could only evaluate the data for colorectal cancer (CRC), stomach, and pancreas cancer groups. Male subjects with CRC had significantly higher expression of miR-133a-3p, -186-5p, -195-5p, -21-5p, 210-3p, -22-3p, -22-5p, -25-3p, -34a-5p, -92a-3p, and -16-5p than females when tested with a *t*-test (Table 1). However, after ANCOVA correction for cancer stage, BMI, and age, only the results for miR-186-5p, -22-3p, 22-5p, 25-3p, -92a-3p, and -16-5p were confirmed (Figure 1C). Whereas miRNA expression of miR-133a-3p and miR-22-5p was significantly lower in men than in women with stomach cancer (Figure 1D), these differences were confirmed after ANCOVA adjustment for cancer stage, BMI, and age (Table 1). No significant differences in miRNA expression were observed between male and female pancreas cancer patients (Supplementary File S1: Sheet S1). The raw Ct values of the miRNAs from Figure 1 are shown in Supplementary File S2: Figure S1. The mean, standard deviation (SD), and standard error of the mean (SEM) of the raw Ct values of all miRNA targets are shown in Supplementary File S3.

### 2.2. DNA Methylation Levels Results

The only difference regarding DNA methylation levels between female and male subjects was observed for *Stratifin* (*SFN*) when analyzing all cancer types combined with an independent *t*-test (*p* = 0.008, Supplementary File S1: Sheet S2). However, after adjustment for cancer type, cancer stage, BMI, and age as covariates, the difference in *SFN* methylation was no longer significant (*p* = 0.072, η^2^ = 0.025). No significant differences in DNA methylation levels of the 12 analyzed targets were observed between male and female subjects for the healthy group, subjects with CRC, stomach, or pancreas cancer (Supplementary File S1: Sheet S2).

### 2.3. Sex-Specific miRNA Expression Related to Cancer Stage

**Figure 2:**
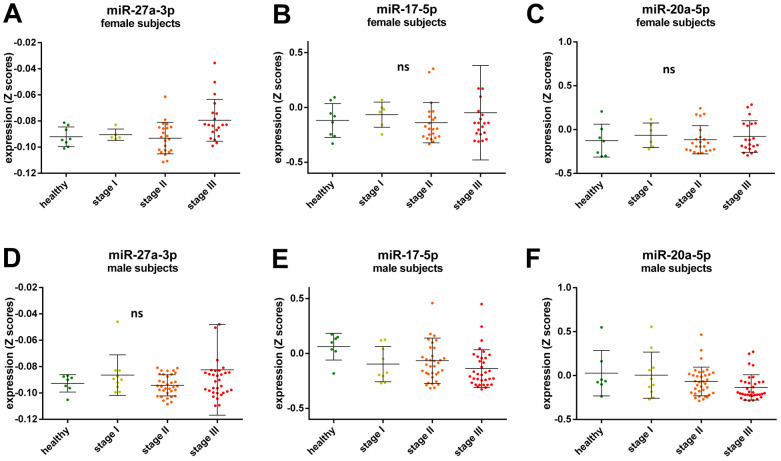
Relative expression of miRNAs in the healthy control group and all cancer samples across cancer stages in female (**A**–**C**) and male subjects (**D**–**F**). The lines in the scatter dot plots represent the mean expression values and the error bars represent the standard deviation (SD). Each dot represents a sample in a specific group as follows: green healthy subjects, yellow subjects with cancer Stage I, orange subjects with cancer Stage II, and red subjects with cancers Stage III; ns not significant.

A Spearman’s rank correlation analysis revealed further sex-specific differences when investigating possible cancer stage related miRNAs. After adjustment for cancer type, BMI, and age a positive correlation with cancer stage for miR-27a-3p expression was observed for female participants (*ρ* = 0.309, *p* = 0.024, Figure 2A), while the expression of miR-17-5p and miR-20a-5p was negatively correlated with cancer stage in the male participants (*ρ* = −0.22, *p* = 0.047 and *ρ* = −0.239, *p* = 0.028 respectively, Figure 2E,F). There were no significant correlations for cancer stage and miR-27a-3p expression in male participants (*ρ* = 0.092, *p* = 0.404, Figure 2D), cancer stage and miR-17-5p (*ρ* = −0.109, *p* = 0.438, Figure 2B), and miR-20a-5p (*ρ* = 0.071, *p* = 0.614, Figure 2C) expression in female participants.

## 3. Discussion

Differential gene expression has been observed between normal healthy tissue and tumor cells, relevant amongst other things to DNA replication and repair, cell cycle, glycolysis, glutaminolysis, and mitochondrial metabolism [22,23,24]. While there are common dysregulated pathways leading to various tumor types, there is a molecular heterogeneity between different cancer types, meaning that their gene regulation and epigenetic traits vary across tumor tissues [25]. Previously, we have reported distinct miRNA expression and methylation levels for specific cancer types [21], now we observe differential DNA methylation and expression of miRNAs between male and female subjects with or without solid tumors. These sex-characteristic epigenetic traits appear to be in contrast depending on the tumor type. The expression of miR-133a-3p and miR-22-5p was higher in female participants with stomach cancer compared to males (Figure 1D). Interestingly, our data showed the exact opposite in colorectal cancer (CRC) subjects, where a higher expression of these two miRNAs was observed in the plasma of male subjects compared to female (Table 1, miR-133a-3p not significant after adjustment). MiR-133a and miR-22 have been described as possible tumor suppressors and metastasis inhibitors [22,26]. However, an oncogenic role of miR-22 has been reported in lung [27] and prostate cancer [28], implying the diverse effects of the same miRNA in different types of cancers. In addition, our results suggest that the role of miR-133a-3p and miR-22-5p could be distinctive not only to the tissue type but also to the biological sex. The potential mechanism of miR-22 in cancer could be explained by its effect on one-carbon metabolism which is crucial for epigenetic maintenance [29]. It has been shown that miR-22 could inhibit the formation of S-adenosylmethionine (SAM) by directly targeting key enzymes in the folate metabolism, methylenetetrahydrofolate dehydrogenase 2 (MTHFD2) and methylenetetrahydrofolate reductase (MTHFR), thereby inducing hypomethylation of tumor suppressor genes and suppressing cancer cell proliferation [29]. The expression levels of enzymes that are involved in the one-carbon metabolism, however, are also affected by sex hormones [30]. Moreover, sex hormones could directly or indirectly regulate miRNA expression [5]. For instance, it has been shown that estrogen binds to regulatory regions of some miRNAs, including miR-21, thereby influencing their expression [31]. Whereas the promoter regions of other miRNAs have responsive elements for testosterone, such as miR-133a/b, proposing a possible mechanism for a sex-specific miRNA regulation [32]. 

Likewise, the dual role of miR-186-5p, both as an oncomir and tumor suppressor, has been discussed in several types of cancers [33]. It has been proposed that this could be explained through the variation of the mRNAs targets of miR-186-5p, for instance, Zinc Finger E-Box Binding Homeobox 1 (*ZEB1*) [34], Family With Sequence Similarity 134 Member B (*FAM134B*) [35], and Deleted In Liver Cancer 1 (*DLC1*) [36]. Our data showed higher miR-186-5p expression in male subjects with CRC than in female patients. Hence, a potential contradictory function of miR-186-5p in biological sexes could be hypothesized. Yet, the difference between miRNA levels could be due to the heterogeneity of tumors or the variety of histological subtypes, as suggested earlier [33]. 

Furthermore, we observed upregulation of several miRNAs in male subjects when compared to females, regardless of cancer type (Figure 1B). Interestingly, miRNA levels in male participants were higher than those in females in the healthy control group, as well as in the CRC group (Figure 1A,C, respectively). This could be a further indication of a dual function of miRNAs depending on the biological sex of the patient. 

Moreover, we found some sex-specific cancer stage-dependent correlations. While an increase of miR-27a-3p was observed with higher tumor stage in female subjects but not in male subjects (Figure 2A,D, respectively), the levels of miR-17-5p and miR-20a-5p decreased with tumor stage in male participants (Figure 2E,F, respectively). MiR-17-5p and miR-20a-5p have been shown to be involved in tumor metastasis and their expression is lower in high metastatic cell lines [37]. Here, we did not analyze samples from patients with metastatic cancers, yet our results indicate that miR-17-5p and miR-20a-5p levels decline significantly with tumor grade, even before the tumor has spread. Therefore, these miRNAs could possibly serve as biomarkers for tumor development in male patients since no significant correlations were observed for female subjects. On the other hand, miR-27a-3p has been identified as an oncogenic miRNA by inhibiting tumor suppressors, and its overexpression is associated with tumor cell proliferation [38]. Thus, miR-27a-3p could be a suitable biomarker for cancer progression in women. 

Interestingly, most of the deregulated miRNAs that we observed had an overall higher expression in male subjects than in female subjects across the control group, CRC group, or all cancer types (Figure 1A–C, respectively). Opposing miRNA expression patterns between the sexes have been previously reported, even for miRNAs from the same family/cluster [39]. Our results showed that miR-17-5p and miR-92a-3p, which belong to the miR-17-92 cluster, were upregulated in male subjects. However, miR-17-5p had opposing expression between the sexes only in healthy subjects, while differential expression for miR-92a-3p was observed in cancer patients. Higher miR-92a expression of male subjects has been previously described for patients with rheumatoid arthritis, but not for the healthy control group [40], thus further indicating the disparate and complex role of miRNAs depending on the health status and sex of the donor. In addition, our data showed sexual dimorphism for miR-16-5p (miR-15 precursor family) plasma levels in male subjects with CRC compared to female CRC patients. 

Besides miRNA, we analyzed the methylation level in 12 DNA CpG-rich promoter regions in the genes *SEPT9*, *MLH1*, *MGMT*, *GATA5*, *GSTP1*, *SFN*, *MDR1*, *VIM*, *SHOX2*, *ALKBH3*, *APC*, and *RASSF1A*. Among them, only one significant difference between female and male participants was observed, namely for *SFN*. The methylation level of *SFN* was higher in men when the data were analyzed from all participants with tumors (Supplementary File S1: Sheet S2). However, after adjustments for covariates, specifically cancer type and stage, body mass index (BMI), and age, the difference in *SFN* methylation was no longer significant. *SFN*, also known as *14-3-3σ*, has been described both as a tumor suppressor gene as well as a tumor oncogene [41]. Sex-dependent DNA methylation patterns have been reported previously in healthy individuals, as well as in relation to diseases [6,42]. Considering the limited number of DNA methylation targets that were analyzed in this study and the relatively small study group, our data are insufficient regarding sex-specific differences in cell-free DNA methylation. A follow-up analysis of additional samples and targets, such as several sites in the promoter region of *SFN*, should be carried out to further evaluate the role of DNA methylation. 

Although this study revealed several interesting results regarding epigenetic differences between males and females, some limitations should be taken into account. Since we analyzed a subset of data that were collected for a previous study [21], we had a relatively small sample size to test this research hypothesis. Moreover, the distribution of men and women between the different study groups was similar only for healthy subjects, subjects with CRC or stomach cancer, or subjects with solid tumors regardless of cancer type (Table 2), thus we could only investigate the statistical differences between men and women in these groups. Despite the small size of these groups, we observed several sex-specific variations in miRNAs expression and with these results we aim to highlight the existence of sex bias in epigenetic cancer biomarkers. Considering that these results are based solely on observational data, we could report only correlations and associations and speculate on the causal relationship between epigenetic biomarkers and sex dimorphism. Ideally, a prospective study with multiple sampling at several time points could improve the understanding of disease progression and the variability between the sexes. Nevertheless, our findings underline the importance of sex-related epigenetic differences when assessing diagnostic and prognostic biomarkers.

## 4. Materials and Methods

### 4.1. Study Population and Sample Processing

For this study, we analyzed a subset of samples with sex-balanced groups that were collected earlier for a previous study [21]. The study was carried out according to the Declaration of Helsinki, and ethical approval was obtained from the local ethics committees. It included samples from 15 individuals without tumors and 204 patients with Stage I, II, or III cancer (bladder, brain, breast, colorectal (CRC), lung, ovarian, stomach, pancreas, prostate, and liver). 

In order to investigate sex-specific biomarkers, for this study, we decided to include cancer types for which we had samples from both female and male donors. Thereby, we excluded breast, ovarian, and prostate cancers from the analysis, while we included samples from patients with bladder, brain, CRC, lung, liver, stomach, and pancreas cancer. Sample collection and processing are described in detail in our previous publication [18]. Shortly, whole blood samples were collected from the participants after obtaining written informed consent, and the plasma was promptly obtained by double centrifugation. Patients’ characteristics are shown in Table 2.

### 4.2. Cell-Free DNA Extraction and Methylation Analysis

Cell-free DNA (cfDNA) was isolated from 4 mL double-centrifuged plasma with a MagMAX™ Cell-Free DNA Isolation Kit (ThermoFisher Scientific, Waltham, MA, USA) in a final volume of 80 µL and stored at −20 °C until further processing. Next, the methylation levels of 12 CpG-rich promoter regions in the genes *SEPT9*, *MLH1*, *MGMT*, *GATA5*, *GSTP1*, *SFN*, *MDR1*, *VIM*, *SHOX2*, *ALKBH3*, *APC*, and *RASSF1A* (Supplementary File S2: Table S1) were analyzed via quantitative real-time PCR (qPCR) as previously described [21]. Briefly, a separation of 70 µL of the purified cfDNA into two fractions—methylated (Me cfDNA) and unmethylated DNA (UnMe cfDNA), was carried out using MethylMiner™ Methylated DNA Enrichment Kit (Invitrogen, ThermoFisher). Consequently, 2 µL of each fraction was amplified and the methylation level of the target region was calculated using a previously published formula [21]:

(1) $$CfDNA\;methylation\;\%=100-(100/\left(1+2^{-{Ct}_{\mathrm{Me}\;\mathrm{cfDNA}\;}-\;{Ct}_{\mathrm{UnMe}\;\mathrm{cfDNA}}}\right).$$

### 4.3. Circulating miRNA Extraction and Analysis

For the miRNA analysis, the total RNA was isolated from 100 µL double-centrifugated plasma with MagMAX™ mirVana™ Total RNA (ThermoFisher) following the manufacturer’s handbook. Consequently, purified miRNA was transcribed into cDNA using the TaqMan™ Advanced miRNA cDNA Synthesis Kit (ThermoFisher), and 48 miRNA targets (Supplementary File S2: Table S2) were quantified via qPCR, as previously published [18]. A total of 15 fmol of *C. elegans* miR-39-3p per sample was used as a spike-in control to adjust for variability during sample processing such as the efficiency of RNA extraction and reverse transcription. Raw Ct values of *C. elegans* miR-39-3p are shown in Supplementary File S2: Figure S2. The global mean expression of all miRNA targets, including *C. elegans* miR-39-3p, was used for data normalization as previously described [43]. Delta Ct values were calculated with the Relative Quantification app on ThermoFisher Connect with the following formula:

(2) ΔCt miRNA=Ct miRNA−((ΣCt all analyzed miRNAs)/48)

For data analysis, the raw miRNA expression values were standardized and converted into z scores, using the following formula:

(3) z score miRNA=(ΔCt miRNA−mean ΔCt miRNA)/SD miRNA

### 4.4. Data Analysis

The statistical data analysis was carried out in IBM^®^ SPSS^®^ Statistics 20 Software. Differences between female and male participants were analyzed with independent *t*-tests and controlled for covariates such as cancer type and stage, body mass index (BMI), and age via ANCOVA or Quade’s test. Effect sizes were measured either via Hedges’ g or partial eta-squared η^2^, after controlling for confounders. Cancer stage-associated correlations were assessed via Spearman’s *ρ* rank coefficient tests and were adjusted for covariates. In addition, an ANCOVA with simple contrast and Bonferroni 95% confidence interval (CI) adjustment for cancer type as a covariate was carried out to investigate differences across healthy participants and cancer Stages I, II, and III. The mean values for miRNA expression and DNA methylation levels in each analyzed group, as well as the *t*-test results, are shown in Supplementary File S1, Sheets S1 and S2, respectively. GraphPad Prism 6 was used to visualize the data and create the figures.

## 5. Conclusions

The recognition that sexual dimorphism is present in various miRNAs and DNA methylation is important for biomarker development in the context of personalized disease management. Studies that focus on the average levels of biomarkers across a specific population frequently do not account for differences between the sexes. While we acknowledge the complexity of miRNAs and their dynamic regulation in various diseases, along with the challenge/impossibility to consider all factors, we believe that it is important to delve deeper into the sexual dimorphism of epigenetics. Exploring the sex differences in carcinogenesis could provide better disease management for both female and male patients and could contribute to the development of personalized medicine.

## Figures and Tables

**Table 1:** Significant *t*-test results between female and male participants in the different groups for miRNA expression and results after adjustments for covariates.

Groups	*n* (f/m)	miRNA	Mean Difference Female–Male (SED)	*t*-Test *p*-Value	Hedges’ g Effect Size	Adjusted *p*-Value	Partial Eta Squared η^2^
Healthy ^c^	15 (8/7)	miR-17-5p	−0.180 (0.072)	0.027	1.291	0.027 *	0.371
		miR-26b-5p	−0.098 (0.042)	0.036	1.208	0.011 *	0.46
All cancer ^a,c^	128 (48/80)	miR-126-3p	−0.352 (0.165)	0.035	0.389	0.017 *	0.046
		miR-21-5p	−0.353 (0.124)	0.005	0.452	0.006 **	0.059
		miR-92a-3p	−0.537 (0.167)	0.002	0.497	0.01 *	0.053
		miR-183-5p	−0.346 (0.168)	0.041	0.318	0.015 *	0.046
		miR-16-5p	−0.269 (0.111)	0.017	0.392	0.075	0.025
CRC ^b,c^	28 (14/14)	miR-133a-3p	−0.558 (0.249)	0.043	0.847	0.076	0.116
		miR-186-5p	−0.463 (0.117)	0.001	1.503	0.003 **	0.287
		miR-195-5p	−0.381 (0.126)	0.007	1.142	0.085	0.11
		miR-21-5p	−0.562 (0.258)	0.039	0.721	0.115	0.104
		miR-210-3p	−0.741 (0.293)	0.018	0.956	0.061	0.145
		miR-22-3p	−0.984 (0.421)	0.027	0.884	0.039 *	0.173
		miR-22-5p	−1.105 (0.407)	0.012	1.025	0.038 *	0.174
		miR-25-3p	−0.431 (0.141)	0.007	1.159	0.003 **	0.324
		miR-34a-5p	−0.496 (0.199)	0.026	0.941	0.411	0.026
		miR-92a-3p	−1.132 (0.423)	0.013	1.013	0.007 **	0.278
		miR-16-5p	−0.644 (0.211)	0.005	1.155	0.021 *	0.21
Stomach cancer ^b,c^	23 (15/8)	miR-133a-3p	0.136 (0.052)	0.019	0.842	0.037 *	0.19
		miR-22-5p	0.823 (0.326)	0.02	1.106	0.042 *	0.21

Adjusted *p*-value and partial eta squared η^2^ are calculated after correction for covariates: cancer type and stage ^a^, only cancer stage ^b^, body mass index, and age ^c^. f female, m male, SED standard error difference, * *p* < 0.05, ** *p* < 0.01.

**Table 2:** Characteristics of the study participants.

Subjects	*n*	Age Years Mean (SD)	BMI kg/m^2^ Mean (SD)	Cancer Stage *n*		
				I	II	III
Female	56	64.0 (14.5)	24.1 (3.4) ^b^	6	22	20
Healthy	8	49.4 (16.7)	25.6 (4.2)	0	0	0
Bladder	3	81.0 (10.8)	23.9 (2.7)	3	0	0
Brain	3	61.3 (17.5)	25.9 (1.5)	1	2	0
CRC	14	69.1 (11.1)	23.7 (2.9)	1	11	2
Lung	4	54.5 (3.0)	24.5 (2.4)	0	1	3
Stomach	15	70.9 (11.2)	23.0 (4.0)	0	5	10
Pancreas	6	62.3 (4.5) ^a^	25.4 (2.1)	0	2	4
Liver	3	47.3 (13.4)	22.7 (5.8)	1	1	1
Male	87	65.3 (9.1)	25.5 (3.5) ^b^	10	35	35
Healthy	7	60.6 (11.5)	25.3 (1.1)	0	0	0
Bladder	17	68.9 (9.1)	26.7 (2.9)	6	6	5
Brain	6	64.2 (9.4)	24.9 (4.1)	1	2	3
CRC	14	64.1 (7.7)	25.6 (2.8)	2	8	4
Lung	25	63.9 (9.4)	29.9 (4.3)	1	11	13
Stomach	8	66.5 (6.8)	22.9 (4.9)	0	3	5
Pancreas	6	71.8 (8.9) ^a^	23.4 (2.5)	0	3	3
Liver	4	61.3 (5.7)	26.3 (0.4)	0	2	2
Total	143	64.8 (11.5)	24.9 (3.6)	16	57	55

Lower case letters indicate a significant difference between female and male participants for age ^a^ and BMI ^b^ at *p* < 0.05 when tested with an independent *t*-test. SD standard deviation, BMI body mass index, CRC colorectal cancer.

## Data Availability

The datasets that were used for the current study are available from the corresponding author on reasonable request.

## Supplementary Materials

The following supporting information can be downloaded at: https://www.mdpi.com/article/10.3390/epigenomes7010002/s1; Supplementary File S1: Sheet S1. miRNAs; Sheet S2. DNA methylation (mean values for miRNA expression and DNA methylation levels and the corresponding *t*-tests results (female vs. male) for all analyzed groups).; Supplementary File S2: Table S1. Genes and regions analyzed for methylation status; Table S2. MiRNA targets; Figure S1. Abundance of miRNAs in plasma samples across the different study groups; Figure S2. Abundance of *C. elegans* miR-39-3p spike-in control across the different study groups.; Supplementary File S3: Mean Ct values, standard deviation (SD), and standard error of the mean (SEM) of all miRNA targets for all the analyzed groups.
