# Supplementary material for: Sex-Specific miRNA Differences in Liquid Biopsies from Subjects with Solid Tumors and Healthy Controls
Source: Epigenomes. 2023 Jan 10;7(1):2. doi: 10.3390/epigenomes7010002 (PMC9844450; doi:10.3390/epigenomes7010002)
Supplement: Supplementary file 1 [file epigenomes-07-00002-s001.zip › Supplementary File 2.pdf]

Sex-specific miRNA differences in liquid biopsies from subjects with solid tumors and healthy controls

Elena Tomeva, Ulrike D. B. Krammer, Olivier J. Switzeny, Alexander G. Haslberger and Berit Hippe

Supplementary file 2: Table S1. Genes and regions analyzed for methylation status.

Gene	NCBI Reference Sequence	Region	Sequence
ALKBH3	NC_000011.10	43880721-43880820	ctacccggac tgaggactgc gcaccggaag tagtggcggc gctgcgtaac gtcgagagga agtgcctgat gccgcggcct gtgattggga gttgctggag
APC	NC_000005.10	112737681-112737780	ctagggctag gcaggctgtg cggttgggcg gggccctgtg ccccactgcg gagtgcgggt cgggaagcgg agagagaagc agctgtgtaa tccgctggat
GATA5	NC_000020.11	62475990-62476090	aagactggaa gcccgggccg ctgaggctcc gcagcccccct ccgcgccgcc ccggcccgcc cccgccgcgc cgccccttcc ctccccgcgc ccgccccttc t
GSTP1	NC_000011.10	67583636-67583735	ctccggggac tccagggcgc ccctctgcgg ccgacgcccg gggtgcagcg gccgccgggg ctggggccgg cgggagtccg cgggaccctc cagaagagcg
MDR1	NC_000007.14	87600483-87600384	ggcagagttg ggggtctggc agcgcgttct ggactttgcc cgccgccagt gcgattctcc ctcccggttc cagtcgccgc ggacgatgct tcctcccacc
MGMT	NC_000010.11	129467232-129467305	cgcccctaga acgctttgcg tcccgacgcc cgcaggtcct cgcggtgcgc accgtttgcg acttggtgag tgtc
MLH1	NC_000003.12	36992887-36993005	tattcgtgct cagcctcgta gtggcgcctg acgtcgcgtt cgcgggtagc tacgatgagg cggcgacaga ccaggcacag ggccccatcg ccctccggag gctccaccac caaataacg
RASSF1A	NC_000003.12	50340772 - 50340673	gccgtgtggg gttgcacgcg gtgccccgcg cgatgcgcag cgcgttggca cgctccagcc gggtgcggcc cttcccagcg cgcccagcgg gtgccagctc
SEPTIN9	NC_000017.11	77373482-77373543	ctgcccacca gccatcatgt cggaccccgc ggtcaacgcg cagctggatg ggatcatttc gg
SFN	NC_000001.11	26863259-26863372	ccgaacgcta tgaggacatg gcagccttca tgaaaggcgc cgtggagaag ggcgaggagc tctcctgcga agagcgaaac ctgctctcag tagcctataa gaacgtggtg ggcg
SHOX2	NC_000003.12	158103869-158103755	cgaccctaaa cgcttaaccc acagagatca acaggttcaa gcggaatatt cgcgatcctc ggtttctatt ggttgctcaa agccttttca tgcaaccagc agctcggatg tttaa
VIM	NC_000010.11	17229166-17229265	gcttctcgct aggtccctat tggctggcgc gctccgcggc tgggatggca gtgggagggg accctctttc ctaacggggt tataaaaaca gcgccctcgg

Supplementary file 2: Table S2. MiRNA targets.

cel-miR-39-3p	hsa-miR-141-3p	hsa-miR-17-5p	hsa-miR-210-3p	hsa-miR-22-5p	hsa-miR-31-5p
hsa-let-7a-5p	hsa-miR-142-5p	hsa-miR-182-5p	hsa-miR-21-5p	hsa-miR-23a-3p	hsa-miR-34a-5p
hsa-miR-101-3p	hsa-miR-143-3p	hsa-miR-183-5p	hsa-miR-218-5p	hsa-miR-25-3p	hsa-miR-375-3p
hsa-miR-106a-5p	hsa-miR-145-5p	hsa-miR-186-5p	hsa-miR-221-3p	hsa-miR-26a-5p	hsa-miR-451a
hsa-miR-1225-3p	hsa-miR-148a-3p	hsa-miR-195-5p	hsa-miR-222-3p	hsa-miR-26b-5p	hsa-miR-497-5p
hsa-miR-124-3p	hsa-miR-148b-3p	hsa-miR-203a-3p	hsa-miR-223-3p	hsa-miR-27a-3p	hsa-miR-92a-3p
hsa-miR-126-3p	hsa-miR-155-5p	hsa-miR-205-5p	hsa-miR-22-3p	hsa-miR-29c-3p	hsa-miR-9-5p
hsa-miR-133a-3p	hsa-miR-16-5p	hsa-miR-20a-5p	hsa-miR-224-5p	hsa-miR-30a-5p	hsa-miR-96-5p

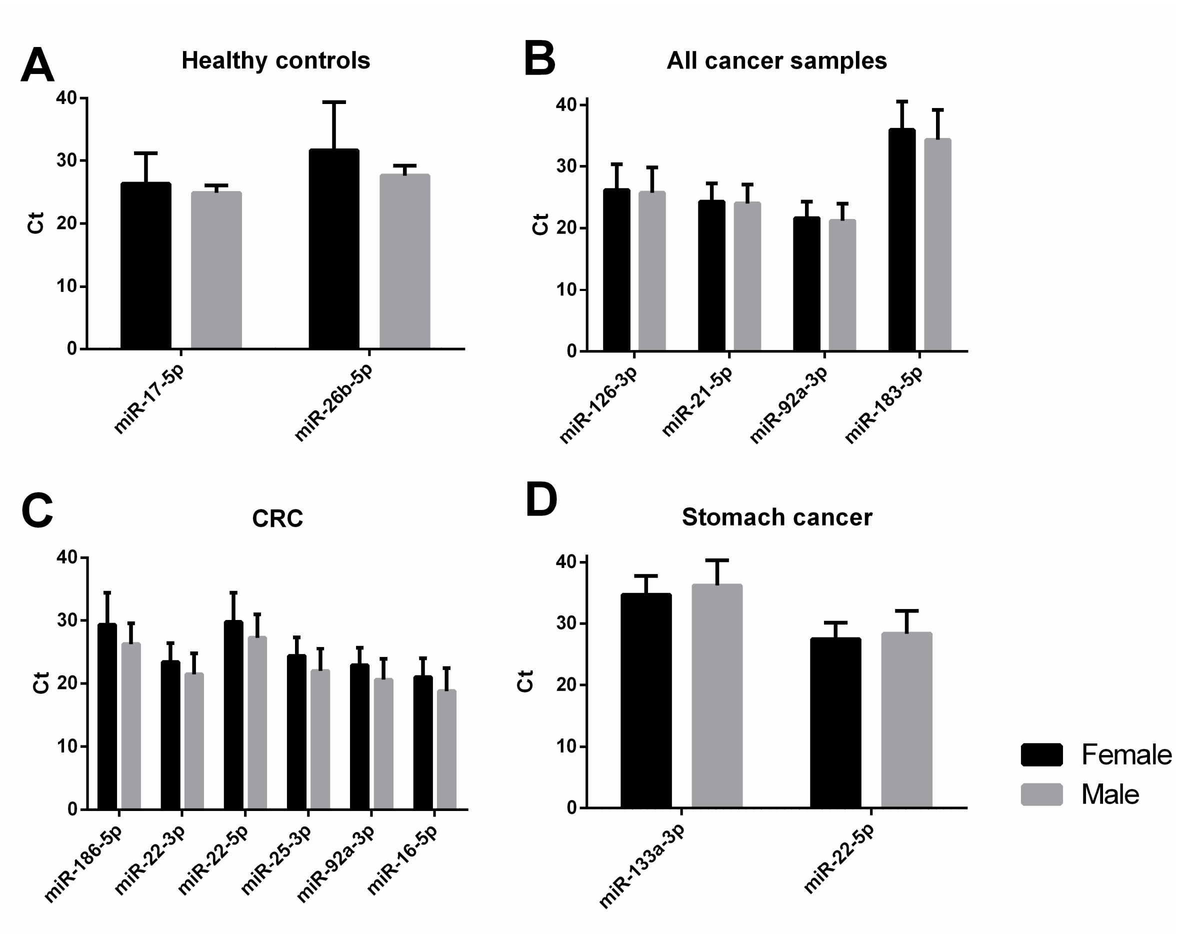

Supplementary file 2: Figure S1. Abundance of miRNAs in plasma samples across the different study groups. Only the mean Ct values of differentially expressed miRNAs between female and male participants (after normalization and adjustments for covariates) are depicted in this figure. Bars represent the mean value with standard deviation (SD). **A** Healthy control group *n* female = 8, *n* male = 7 **B** All cancer samples, *n* female = 48, *n* male = 80 **C** Colorectal cancer group, *n* female = 14, *n* male = 14 **D** Stomach cancer, *n* female = 15, *n* male = 8

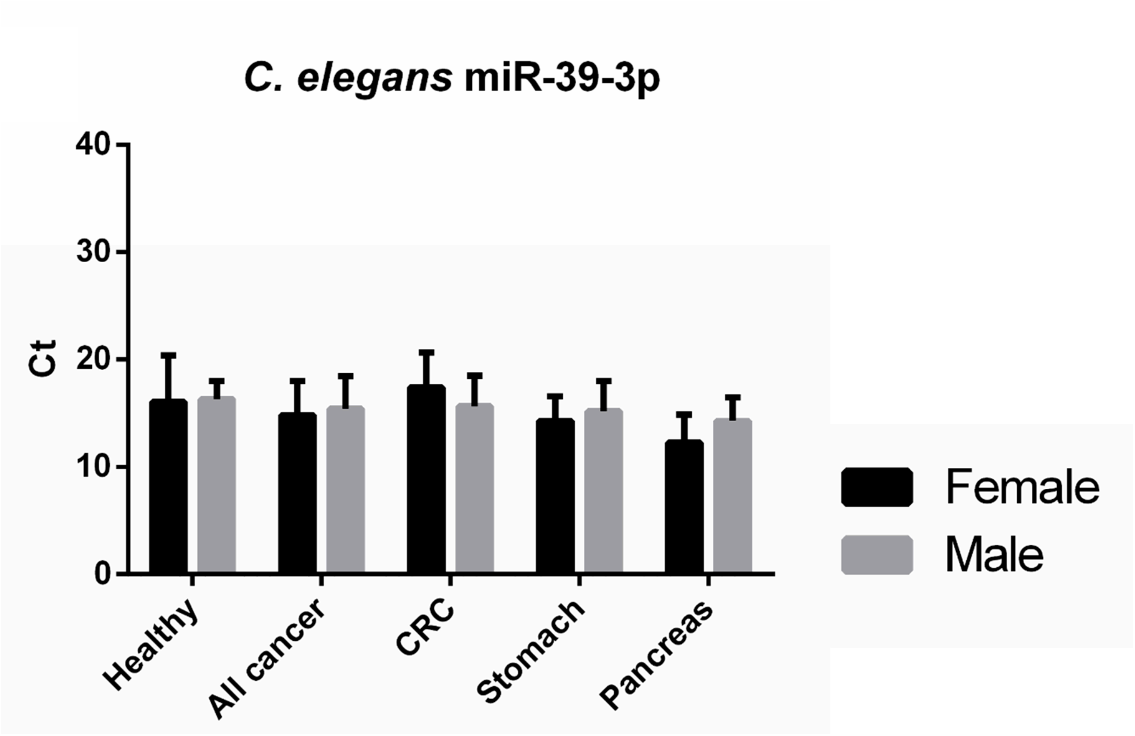

Supplementary file 2: Figure S2. Abundance of *C. elegans* miR-39-3p spike-in control across the different study groups. 15 fmol of C. elegans miR-39-3p was added to each sample during RNA extraction. Bars represent the mean Ct value with SD.
